# Exposure to Ambient Heat and Kidney Function in the Health and Retirement Study

**DOI:** 10.1093/geroni/igaf122.794

**Published:** 2025-12-31

**Authors:** Kristina Van Dang, Eun Young Choi, Eileen Crimmins, Jennifer Ailshire

**Affiliations:** University of Southern California, Los Angeles, California, United States; University of Southern California, Los Angeles, California, United States; University of Southern California, Los Angeles, California, United States; University of Southern California, Los Angeles, California, United States

## Abstract

Increased heat events are one of the defining characteristics of climate change affecting human health. The kidney’s role in homeostasis makes them particularly susceptible to heat, especially in older populations in which kidney function is declining. Using data from the Health and Retirement Study (HRS) Venous Blood Study (N = 9,933), we linked respondents to census tract level daily heat index from 7 days prior to blood collection. Kidney function was assessed with estimated glomerular filtration rate based on serum creatinine (eGFRcr). The average age of our analytic sample was 69 years (SD = 10). About 18% of the sample would be classified as having chronic kidney disease based on an eGFRcr < = 60 mL/min/1.83m2. Using linear models with cluster-adjusted standard errors, and controlling for age, gender, race/ethnicity, education, and urbanicity, we found that each unit increase in prior 7-day average heat-index was associated with a decreased eGFRcr of 0.04 (95% CI: -0.06, -0.02). We also categorized heat index days into cautionary (heat index > 80, the level at which the National Weather Service reports fatigue is possible) and extreme cautionary levels (heat index>90, the level at which NWS reports sunstroke, muscle cramps, and/or heat exhaustion is possible). We found that more days at cautionary level was associated with -0.96 lower (95% CI: -2.0, 0.05) eGFRcr and more days at extreme cautionary levels was associated with -2.5 lower (95% CI: -3.7, -1.3) eGFRcr. Our findings suggest kidney function in older adults can be adversely affected by exposure to short-term high heat.

